# Proportion of Chromosomal Disorders and Their Patterns among Births with Congenital Anomalies in Africa: A Systematic Review and Meta-Analyses

**DOI:** 10.1155/2022/6477596

**Published:** 2022-12-13

**Authors:** Teshome Gebremeskel Aragie, Girma Seyoum Gedion

**Affiliations:** ^1^Woldia University, College of Health Sciences, Department of Anatomy, Woldia, Ethiopia; ^2^Addis Ababa University, College of Health Sciences, School of Medicine, Department of Anatomy, Addis Ababa, Ethiopia

## Abstract

**Introduction:**

Worldwide, surveys have shown that the frequency of chromosomal disorders among births with congenital anomalies varies greatly from country to country. It is well known that chromosomal disorders are an important cause of premature death or life-long disability; however, the absence of local epidemiological data on their birth prevalence and outcomes impedes policy and service development in many countries and continents. Therefore, the current systematic review and meta-analysis intend to show the pooled proportion of chromosomal disorders among births with congenital anomalies in Africa.

**Methods:**

From PubMed, Cochrane Library, and Google Scholar, we systematically reviewed and meta-analyzed the studies that examined the incidence, prevalence, and types of chromosomal disorders using PRISMA guidelines. A weighted inverse variance random-effects model was used to estimate the pooled proportion of chromosomal disorders among births with congenital anomalies.

**Results:**

From the total of 3,569 studies identified, 1,442 were from PubMed, 108 were from Cochrane Library, 1,830 were from Google Scholar, and 189 were from other sources. After duplication was removed, a total of 844 articles remained (2725 were removed by duplication). Finally, 144 full-text studies were reviewed and 60 articles with 52,569 births having congenital anomalies met the inclusion criteria and were selected for this meta-analysis. The pooled proportion of chromosomal disorders among births with congenital anomalies was 8.94% (95% CI; 7.02, 10.86; *I*^2^ = 98.8%; *p* < 0.001). *Conclusions and Future Implications*. In the current systematic review and meta-analysis, the pooled proportion of chromosomal disorders among births with congenital anomalies in Africa was small. Down syndrome (trisomy 21) accounted for more than 80% of chromosomal disorders. The pooled proportion of chromosome disorders was the highest in North African regions and countries compared to other regions of the continent. Healthcare managers should focus on establishing proper cytogenetic diagnostic facilities in collaboration with well-trained genetic counseling services in the continent.

## 1. Introduction

Chromosomal anomalies are the leading causes of congenital anomalies, whether inherited or newly discovered [1, 2]. Worldwide, among the diverse causes of congenital anomalies, genetic/chromosomal disorders are still on a disturbing rise, which leads to a major health problem; indeed, critical control of environmental diseases has been realized [3]. Nowadays, with the enduring control of communicable diseases and malnutrition, chromosomal disorders associated with congenital anomalies are making a relatively noticeable impact on poor childhood health [4]. Available evidence suggests that congenital and genetic disorders are responsible for a major proportion of infant mortality, morbidity, and handicap and are 20% higher in sub-Saharan Africa than in industrialized countries [5]. Chromosomal disorders can occur in any pregnancy sporadically, but the risk of having a pregnancy affected by T21, T13, or T18 is known to increase with maternal age [6–9]. Recently, environmental factors such as air pollution and proximity to hazardous waste sites have been reported to increase the risk of structural birth defects and chromosomal disorders [3, 4].

In low-income/middle-income countries like Africa, they are likely to be underestimated due to the absence of accurate cause of death data [10, 11]. It has a huge impact on general health and wellbeing, causing multiple problems including either mental retardation and/or physical disabilities, especially in low-income countries. The disorders, especially, aneuploidy in the conceptus or fetus, occur in 5–10% of all pregnancies and are the most common reproductive problem in human beings [12]. Most embryos with chromosomal disorders die in utero, resulting in early pregnancy loss [13]. Cytogenetic evaluation of spontaneous abortions has shown that 50–70% are chromosomally abnormal [14, 15]. Although disorders of the sex chromosomes have a lesser effect on survival, they can cause infertility and congenital malformations such as congenital heart disease [16], as well as have neurodevelopmental and psychological impacts [16–18].

Most autosomal disorders cause death before the age of 5 years or are characterized by multi-domain disability. Despite the treatment and rehabilitation of children with these disorders being costly and complete recovery being usually impossible [19], the availability of appropriate medical care in high-income settings is responsible for fewer deaths before the age of five years [20–22].

In fact, chromosomal disorders are an important cause of premature death or life-long disability; however, the absence of local epidemiological data on their birth prevalence and outcomes impedes policy and service development in many countries and continents [23]. Besides this, advances in the knowledge of the causal pathway leading to congenital anomalies can be the basis for better primary prevention interventions, resulting in longer and better lives. For clinicians and parents, it is important to understand what can be done today to prevent congenital anomalies, in particular the role of preconception care focusing on optimal women's health (including screening/treating chronic illnesses, etc.). In addition, investigation of potential causes of congenital anomalies at the time of diagnosis (such as when a genetic condition is present) can help to better plan management and appropriately counsel families, including the relief of anxiety related to unfounded information and fault [24].

Although there are perinatal studies on the birth prevalence of congenital anomalies in different regions of Africa till now, only limited data are available on the birth prevalence of chromosomal disorders in births with congenital anomalies. To the best of our knowledge and search, there is a paucity of data on the pooled proportion and pattern of chromosomal disorders in Africa. Thus, the main aim of the present systematic review and meta-analysis was to estimate the pooled proportion and patterns of the chromosomal disorders in the African continent. Therefore, the result of this meta-analysis and systematic review will help stakeholders, policymakers, and concerned bodies in planning and implementing strategies.

## 2. Methods

### 2.1. Review Question

The review question of this systematic review and meta-analysis was as follows:

What are the pooled proportion and patterns of chromosomal disorders in the African context?

### 2.2. Study Selection and Screening

Endnote version 8 reference managers were used to remove duplicated studies after retrieval of the studies. Two investigators (TG and GS) autonomously screened the selected studies using the article's title and abstracts before retrieval of full-text papers. The authors used pre-specified inclusion criteria to further screen the full-text articles. Disagreements were resolved during a consensus meeting for the final selection of studies to be included.

### 2.3. Inclusion and Exclusion Criteria

In this systematic review and meta-analysis, the authors included cross-sectional, case-control, RCT, and cohort studies conducted on populations residing in Africa that reported the prevalence, proportion, incidence, and patterns of chromosomal disorders among births with congenital anomalies in Africa or had enough data to compute these estimates. Studies that reported the prevalence of at least one chromosomal disorder published in the English language from January, 2000 to October, 2021 were included in this meta-analysis. Citations without abstract and/or full-text, anonymous reports, editorials, letters, commentaries, reviews, and qualitative studies were excluded from the analysis. In addition, studies conducted among populations of African origin but residing outside the continent and studies without advanced diagnostic tools (cytological and detailed clinical) for confirmation of chromosomal disorders (e.g., suspected but nonconfirmed disorders) were excluded from this review and analysis. Articles that were not retrievable from the internet and did not report an outcome of interest were excluded after a detailed review of their full texts.

 
**Study area**: Only articles conducted on people living in the African continent.
 
**Study design**: Observational studies (cross-sectional and case-controls and cohort) and RCTs that contain original data reporting the prevalence, incidence, and patterns of chromosomal disorders among births with congenital anomalies in Africa were considered.
 
**Language**: Articles published in the English language were included.
 
**Population**: Studies conducted on congenital anomalies/birth defects which reported chromosomal disorders were counted in.
 
**Publication condition**: Both published and unpublished articles that reported incidence, prevalence, and patterns of chromosomal disorders among births with congenital anomalies in the African continent were considered.

### 2.4. Search Strategy

This review identified studies that provide data on the prevalence, incidence, and patterns of chromosomal disorders among births with congenital anomalies in the context of Africa. In the search engine, mainly PubMed, Google Scholar, and Cochrane library were retrieved. The search included keywords that are combinations of PICO (population, intervention, condition/context, and outcome). A snowball search of the references of all relevant papers for linked articles was also performed. The search terms or phrases included were as follows: “intrauterine,” “birth,” “newborn,” “infant,” “congenital anomalies,” “birth defects,” “congenital disorders,” “congenital malformations,” “chromosomal disorders,” “chromosomal aberrations,” “Down syndrome,” “Edwards' syndrome,” “Patau syndrome,” and “Africa”. Using these key terms, the following search map was applied: (prevalence OR magnitude OR incidence OR pattern) AND (birth (MeSH Terms) OR intrauterine OR infant) AND (congenital anomalies (MeSH Terms) OR birth defects (MeSH Terms) OR congenital disorders (MeSH Terms) OR congenital malformations (MeSH Terms) OR chromosomal disorders (MeSH Terms) OR Down syndrome (MeSH Terms) OR Edwards' syndrome (MeSH Terms) OR Patau syndrome (MeSH Terms) OR Turner syndrome (MeSH Terms) AND African countries on PubMed database. Thus, the PubMed search combines ^#^1 AND ^#^2 AND ^#^3. These search terms were further paired with the names of each African country. On both Cochrane Library and Google Scholar, a built-in text search was used in the advanced search section of the sources.

### 2.5. Quality Assessment

The Newcastle–Ottawa scale assessment tool, as modified for observational studies, was used by the researcher to assess the quality of the papers included in this systematic review and meta-analysis [25]. The tool is divided into three main sections that are intended to evaluate methodological quality, study comparability, and original article quality for statistical analysis. Each original article's quality was assessed by the researcher using the tool as a checklist. Finally, articles that met at least 50% of the quality assessment criteria were deemed appropriate for the analysis [25].

### 2.6. Data Extraction

The country, year of publication, study design, prevalence/incidence, and patterns of chromosomal disorders among births with confirmed congenital defects were taken into consideration by the authors while they created a data extraction form on the Excel sheet. Using four papers chosen at random, the data extraction sheet was tested and then modified. Together, the authors used the extraction form to extract the data. The accuracy of the data was independently verified by the authors as well. Any discrepancies between reviewers were settled through point-by-point talks as necessary. Cross-referencing the data with the accompanying publications allowed the authors to fix the data's typos [26].

### 2.7. Synthesis of Results

The authors exported the data to STATA 14 for analysis after they were was extracted in an Excel sheet. The authors pooled the overall proportion estimates of chromosomal disorders and their patterns (Down syndrome, Edwards' syndrome, Patau syndrome, Turner syndrome, chromosomal deletions, and all other unclassified chromosomal disorders) using a random effect meta-analysis model. The authors conducted the heterogeneity of effect size using *Q* statistics and the *I*^2^ statistics for the general proportion of chromosomal disorder. In this study, the *I*^2^ statistic value of zero indicates true homogeneity, whereas the values of 25, 50, and 75% represented low, moderate, and high heterogeneity, respectively [27]. Subgroup analysis was done by the study country, region of the continent, study design, and year of publication. Sensitivity analysis was employed to examine the effect of a single study on the overall proportion estimation. Publication bias was checked by a funnel plot and more objectively through Egger's regression test [28].

## 3. Results

**Figure 1:**
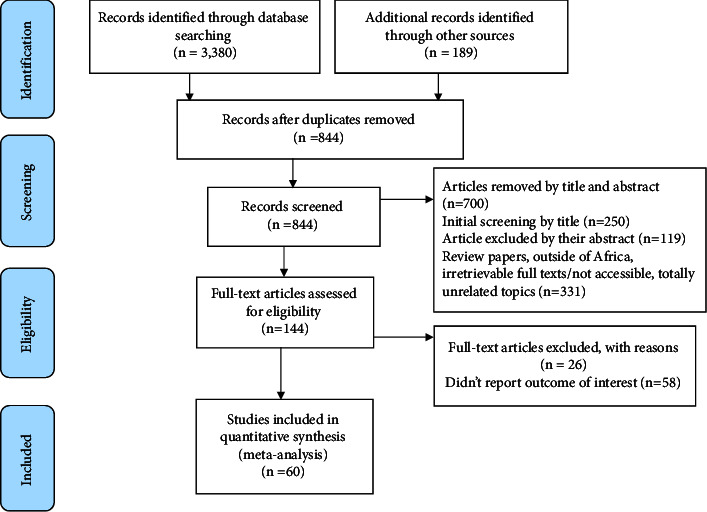
PRISMA-adapted flow diagram showing the results of the search and reasons for exclusion [29].

A total of 3,569 studies were identified; 1,442 were from PubMed, 108 were from Cochrane Library, 1,830 were from Google Scholar, and 189 were from other sources. After duplication was removed, a total of 844 articles remained (2725 were removed by duplication). Finally, 144 full-text articles were reviewed and 60 articles with 52,569 congenital anomaly cases met the inclusion criteria and were selected for this meta-analysis (Figure 1).

### 3.1. Characteristics of Included Studies

**Figure 2:**
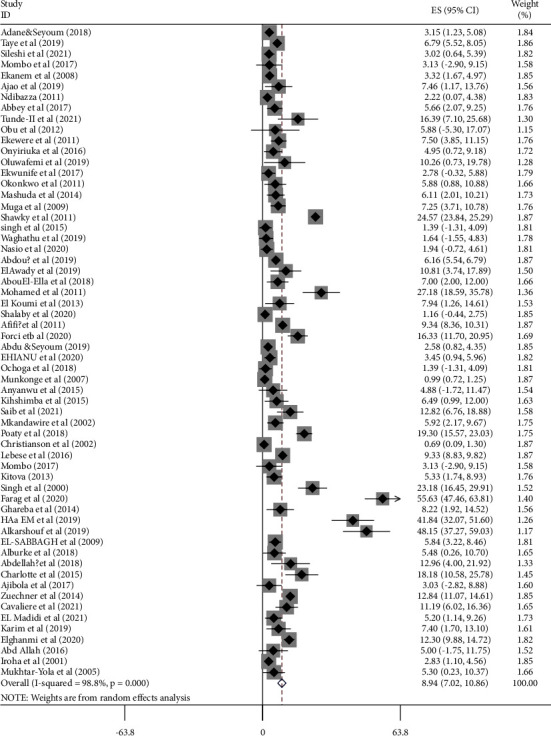
Forest plot on the pooled proportion of chromosomal disorders among births with congenital anomalies in Africa from January, 2000 to October, 2021.

In this systematic review and meta-analysis, 60 articles, which included about 3,091,946 births and 52,569 congenital anomalies, were analyzed. Of these, 4 studies were conducted in Ethiopia [30–33]; 16 studies were conducted in Nigeria [34–48]; 9 studies were conducted in Egypt [49–59]; 3 studies were conducted in South Africa [60–64]; 5 studies were conducted in Morocco [65–69]; 6 studies were conducted in Libya [70–75]; 2 studies were conducted in Tunisia [76–79]; and 7 studies were conducted in Mozambique [80], Botswana [81], Cameroon [82], the DRC [83], Malawi [84], Zambia [85], Gabon [86], and Uganda [87]. Two studies were from Sudan [88, 89]. Three studies were from Tanzania [90–92] and the remaining 3 studies were conducted in Kenya [93–95]. Based on the study design used, 2 studies were conducted by the case-control study design, 3 studies were conducted by cohort study, and 1 study was conducted by a randomized control trial; in contrast, the remaining 54 studies were conducted by the cross-sectional study design. Thirty-nine (65%) studies were published between 2015 and 2021 and the remaining 21 (35%) were published between January 2000 and December 2014. The total number of births/participants with congenital anomalies in the included studies ranged from 17 [38] to 13,543 [49] (Figure 2, Table 1).

## 4. Meta-Analyses

### 4.1. Chromosomal Disorders

#### 4.1.1. Proportion of Chromosomal Disorders

All studies (*n* = 60) included in this review and meta-analysis have reported the prevalence of chromosomal disorders among births with congenital anomalies. Therefore, the pooled proportion of chromosomal disorders ranged from 0.69 [61] to 55.6 [71]. The random-effects model analysis from those studies revealed that the pooled proportion of chromosomal disorders among births with congenital anomalies in Africa was 8.94% (95% CI; 7.02, 10.86; *I*^2^ = 98.8%; *p* < 0.001) (Figure 2).

#### 4.1.2. Subgroup Analysis for the Pooled Proportion of Chromosomal Disorders among Births with Congenital Anomalies in Africa

**Figure 3:**
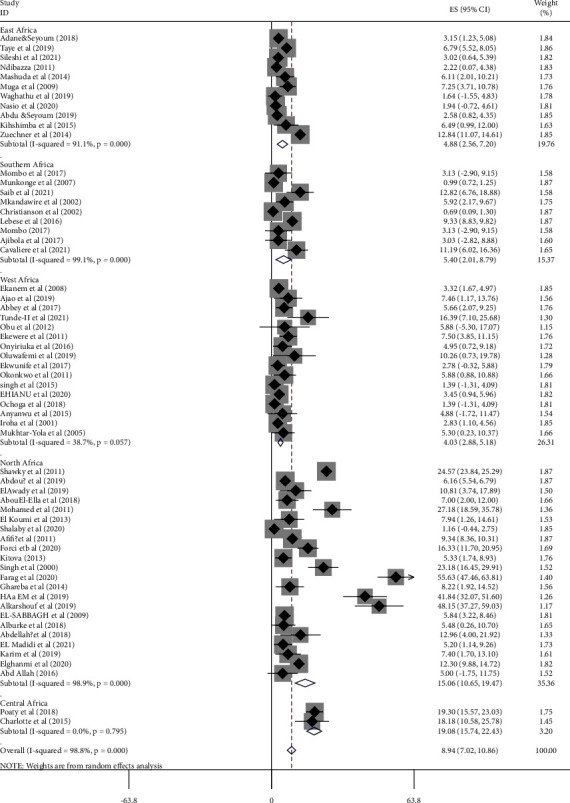
Forest plot on the pooled proportion of chromosomal disorders among births with congenital anomalies in Africa from January, 2000 to October, 2021.

The subgroup analysis was conducted stratified by the region of the continent, country, study design, and year of publication. Based on region of the continent, the pooled proportion of chromosomal disorders among births with congenital anomalies was 19.08% (95% CI: 15.74, 22.43: *I*^2^ = , *p* < 0.79) in Central African countries, 15.06% (95% CI: 10.65, 19.47: *I*^2^ = 98.9, *p* < 0.001) in North African countries, 5.4.0% (95% CI: 2.01, 8.79: *I*^2^ = 99%, *p* < 0.001) in Southern African countries, 4.880% (95% CI: 2.56, 7.20: *I*^2^ = 91.1%, *p* < 0.001) in East African countries, and 4.03% (95% CI: 2.88, 5.18: *I*^2^ = 38.7%, *p*=0.05) in West African countries (Figure 3, Table 2).

**Figure 4:**
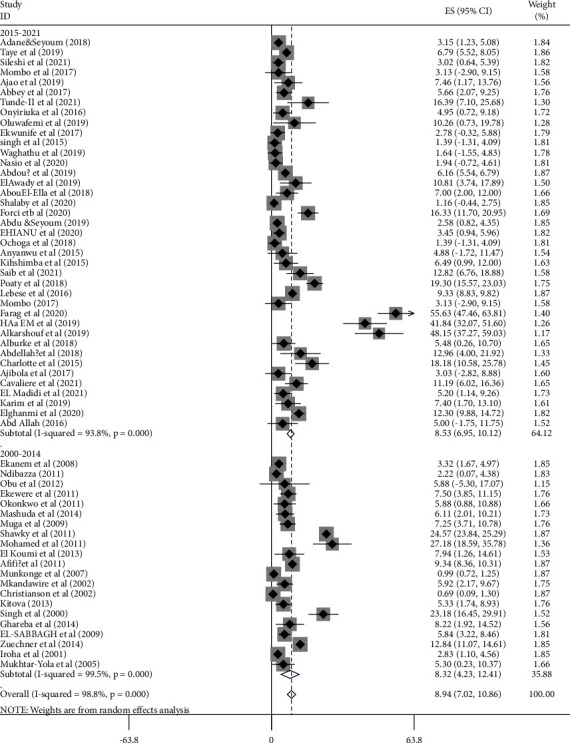
Forest plot on the pooled proportion of chromosomal disorders among births with congenital anomalies in Africa from January, 2000 to October, 2021.

Based on the year of publication, the proportion of chromosomal disorders among births with congenital anomalies in Africa was 8.32 (95% CI: 4.23, 12.15: *I*^2^ = 99.5%, *p* < 0.001) among studies conducted from January, 2000 to December, 2014, while it was 8.53% (95% CI: 6.95, 10.12: *I*^2^ = 93.8%, *p* < 0.001) among studies conducted from January, 2015 to October, 2021 (Figure 4, Table 3).

### 4.2. Heterogeneity

#### 4.2.1. Sensitivity Analysis

To identify the influence of individual studies on the pooled proportion of chromosomal disorders, leave-one-out sensitivity analysis was employed; the results of this sensitivity analysis showed that the current findings were not dependent on a single study. The pooled estimated proportion of chromosomal disorders varied between 8.05 [49] and 9.14 [61] after the deletion of a single study (Figure S1).

#### 4.2.2. Publication Bias

**Figure 5:**
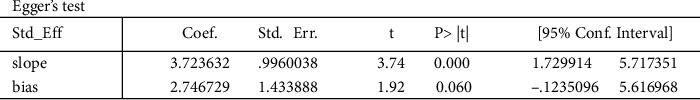
Egger's test to show publication bias on the pooled proportion of chromosomal disorders among births with congenital anomalies in Africa from January, 2000 to October, 2021.

A funnel plot revealed a distribution that was just barely symmetrical. Egger's regression test value was 0.060, indicating that publication bias was not present (Figure 5, Figure S2).

### 4.3. Patterns of Chromosomal Disorders in Africa

#### 4.3.1. Down Syndrome (Trisomy 21)

From the total of 60 included studies, forty-eight (*n* = 48) studies have reported the prevalence of Down syndrome (Trisomy 21) among births with congenital anomalies. The pooled proportion of Down syndrome ranged from 0.45% [85] to 48.1% [74]. The random-effects model analysis from these studies revealed that the pooled proportion of down syndrome among births with congenital anomalies in Africa was 7.67% (95% CI: 5.7, 9.63; *I*^2^ = 98.9.5%; *p* < 0.001) (Figure S3).

#### 4.3.2. Edwards' Syndrome (Trisomy 13)

In this review and meta-analysis, from the total of 60 included studies, sixteen (*n* = 16) studies have reported the prevalence of Edwards' syndrome (Trisomy 18) among births with congenital anomalies. The pooled proportion of Edwards' syndrome ranged from 0.31% [49] to 4.1% [75]. The random-effects model analysis from those studies revealed that the pooled proportion of Edwards' syndrome among births with congenital anomalies in Africa was 0.94% (95% CI: 0.49, 1.40; *I*^2^ = 31.2%; *p*=0.11) (Table 3, Figure S4).

#### 4.3.3. Patau Syndrome (Trisomy 18)

Among the 60 articles included in this review and meta-analysis, only eleven (*n* = 11) have reported the prevalence of Patau syndrome (Trisomy 13) among births with congenital anomalies. The pooled proportion of Patau syndrome ranged from 0.26% [49] to 5.8% [38]. The random-effects model analysis from those studies revealed that the pooled proportion of Patau syndrome among births with congenital anomalies in Africa was 0.92% (95% CI: 0.34, 1.51; *I*^2^ = 49.7%; *p* < 0.03) (Figure S5).

#### 4.3.4. Turner Syndrome

In the current review and meta-analysis, among the 60 included studies, only five (*n* = 5) have reported the prevalence of Turner syndrome among births with congenital anomalies. The pooled proportion of Turner syndrome ranged from 0.97% [93] to 4.85% [52]. The random-effects model analysis from these studies revealed that the pooled proportion of Turner syndrome among births with congenital anomalies in Africa was 1.50% (95% CI: 1.30, 1.71; *I*^2^ = −; *p* < 0.5) (Table 3, Figure S6).

#### 4.3.5. Chromosomal Deletions

Eight (*n* = 8) out of 60 included studies have reported the prevalence of chromosomal deletions among births with congenital anomalies. The pooled proportion of chromosomal deletions ranged from 0.35% [85] to 3.0% [50]. The random-effects model analysis from these studies revealed that the pooled proportion of chromosomal deletions among births with congenital anomalies in Africa was 0.92% (95% CI: 0.37, 1.47; *I*^2^ = 82.3%; *p* < 0.001) (Figure S7).

#### 4.3.6. Unclassified Chromosomal Disorders

In this systematic review and meta-analysis, the authors merged “others” and unclassified chromosomal anomalies” into “unclassified chromosomal disorders.” Ten studies (*n* = 10) out of 60 included articles were reported the prevalence of unclassified chromosomal disorders among births with congenital anomalies. The pooled proportion of unclassified chromosomal disorders ranged from 0.81% [65] to 8.1% [48]. The random-effects model analysis from these studies revealed that the pooled proportion of unclassified chromosomal disorders among births with congenital anomalies in Africa was found to be 1.78% (95% CI: 0.97, 2.60; *I*^2^ = −; *p*=0.36) (Figure S8).

## 5. Discussion

Congenital anomalies are a global problem, but their impact is particularly severe in middle- and low-income countries where more than 94% of the births with serious congenital anomalies and 95% of the deaths of these children [96]. This systematic review and meta-analyses identified the proportion of chromosomal disorders among births with congenital anomalies, highlighting the extent of this serious and vastly underappreciated public health problem in Africa.

In the current review, the pooled proportion of chromosomal disorders in Africa was 8.94 (CI: 7.02, 10.86: *I*^2^: 98.8%; *p* < 0.001). The result of this meta analyses is comparable with a large-scale study conducted in Bulgaria: in which the proportion of chromosomal disorders was 8.2% among births with congenital anomalies [97]. The current result is higher compared to the study conducted in industrialized nations, in which only 6% of chromosomal disorders accounted for among births with congenital anomalies [98]. The reason might be due to the sharp differences in maternal health and other significant risk factors, including poverty, a high percentage of older mothers, and a greater frequency of consanguineous marriages in low- and middle-income countries [99]. However, the current result was lower compared to the large population-based study conducted in Atlanta, USA, in which 12.3% of births had a chromosomal abnormality among those with congenital heart diseases [100], The difference implies that, due to limited access to family planning and deficient or absent prenatal cytogenetic screening, diagnosis, and associated services in developing countries; local registry data are also low compared to developed countries [99], and in developed regions where cytogenetic technology advances and more discoveries are made on the genetic causes of birth defects, the proportion of congenital anomalies with a known cause would be increased [24]. On the other hand, possible investigation and recording of the cause of congenital anomalies is more likely for live births than terminated or dead embryos, particularly where there is the widespread use of private maternity services or termination of pregnancy is illegal or a highly politicized issue. Therefore, the combined effects of these scenarios could be the cause of the reported total birth prevalence being substantially lower than predicted and the reported proportion of terminations and stillbirths being substantially lower than the expected proportion of chromosomal disorders in Africa.

The current meta-analysis result was also lower than the large-scale population-based study conducted by Utah's population-based surveillance in the USA, where chromosomal disorders accounted for 15.3%, out of the total congenital anomalies [24]. In developing countries, due to the absence/deficiency of advanced cytogenetic investigation, most cases of chromosomal anomalies are missed; the resulting underestimation of the burden of disease can have serious policy implications and hinder investments in research and interventions for better prevention and treatment of these major threats to childhood survival and life-long health. Based on country, the pooled proportion of chromosomal disorders was 30.12% (95% CI: 13.17, 47.07) in Libya compared to other countries in the continent. The reason might be due to the high prevalence of consanguineous marriage; the survey of family clinic data showed that 37.6% of all marriages were intrafamilial in the city of Benghazi, Libya [101]. The high prevalence of consanguineous marriage resulted in the higher expression of dominant and recessive genetic disorders [5, 102, 103]. On the other hand, in this meta-analyses, the regional proportion of chromosomal disorders among births with congenital anomalies was the highest in central African countries 19.08% (95% CI: 15.74, 22.43), the pooled result was analyzed by only the two available studies from the region. Therefore, the pooled proportion of chromosomal disorders in North African countries was plausibly higher at 15.06% (95% CI: 10.65, 19.47: *I*^2^; 98.8%, *p* < 0.001) analyzed by incorporating 22 available studies from the region. In this case, the possible suggestion might also go to the dominance of consanguineous marriage (22% in Algeria, 35.5% in Egypt, 37.6% in Libya, 47.2% in Mauritania, 19.9% in Morocco, and 38% in Tunisia) in the region [104].

Based on the year of publication, in the current meta-analyses, the pooled proportion of chromosomal disorders was higher among the studies conducted from 2015 to 2021 than the studies published from 2000–2014. The possible reason might be due to the advancement and increase in attention given to determining the cause of congenital anomalies.

In the current systematic review and meta-analyses, Down syndrome (trisomy 21) was the most common chromosomal disorder reported; the pooled proportion among births with congenital anomalies was 7.6% (95% CI: 5.70, 9.67). The result was supported by the studies conducted in the USA, which reported that Down syndrome is the most commonly identified genetic form of mental retardation and the leading cause of specific birth defects and medical conditions [105], and it was in line with the studies conducted in the Arab world [1, 106, 107] and Europe [108].

### 5.1. Strength and Limitations

As a strength, the authors used a prespecified protocol for search strategy, data abstraction, and quality assessment. The included studies were at low risk of bias based on the Newcastle–Ottawa quality assessment checklist. Moreover, the authors employed subgroup analysis based on study country, study design, and year of publication and sensitivity analysis to identify the small study effect and the risk of heterogeneity. Nevertheless, there may be a publication bias because not all pieces of grey literature, unpublished, and non-retrievable articles are included. Another foreseeable limitation of this meta-analysis is that it does not really cover the entire African continent and population. In this meta-analyses, the absence and scarcity of studies from each country are the main limitations. In addition, language bias was also a limitation, since all the included studies were published in English.

## 6. Conclusions

Based on the current meta-analyses, the pooled proportion of chromosomal disorders in Africa is small. Regarding its patterns, Down syndrome accounted for a higher proportion than other chromosomal disorders. The pooled proportion of chromosome disorders was the highest in North African countries and regions compared to other regions of Africa.

### 6.1. Recommendations and Future Implications

Healthcare managers should establish proper cytogenetic diagnostic facilities in collaboration with well-trained genetic counseling services to provide information and increase community awareness.

Continued recording and investigation of all children with congenital anomalies should be part of an effort to establish the true figures, and this helps define the extent of the problem and helps with policy-making, program prioritization, and resource allocation.

The current review and meta-analyses highlighted the large gaps in the reported and the expected proportion of chromosomal disorders among births with congenital anomalies. This gap in turn could be an opportunity for both basic and public health researchers, which can be very powerful if conducted by combining surveillance programs enriched with clinical and cytogenetic expertise for better case identification.

## Figures and Tables

**Table 1:** Distribution of included studies on the proportion and patterns of chromosomal disorders among births with congenital anomalies in Africa, from January 2000 to October 2021.

Author	Year	Country	Design	Sample size	No of cases	Chrom prop	Down T-21	Edwards' T-18	Patau T-13	Un-class	Turner	CHR Delet
Adane & Seyoum	2018	Ethiopia	CRS	19650	317	3.15	3.15					
Taye et al.	2019	Ethiopia	CRS	76201	1518	6.78						
Sileshi et al.	2021	Ethiopia	CRS	3346	199	3.0	3.01					
Mombo et al.	2017	Gabon	CRS	3500	32	3.1	3.1					
Ekanem et al.	2008	Nigeria	CRS	127,929	452	3.3	3.31					
Ajao et al.	2019	Nigeria	CRS	1057	67	7.4	7.5					
Ndibazza	2011	Uganda	RCT	2365	180	2.2	2.2					
Abbey et al.	2017	Nigeria	CRS	7670	159	5.6	2.51	3.14				
Tunde-II et al.	2021	Nigeria	CRS	502	61	16.3	8.19			8.1		
Obu et al.	2012	Nigeria	CRS	607	17	5.8			5.8			
Ekewere et al.	2011	Nigeria	CRS	200	200	7.5	1	2	2	2.5		
Onyiriuka et al.	2016	Nigeria	CRS	13,858	101	4.9	2.97			1.98		
Oluwafemi et al.	2019	Nigeria	CRS	39	39	10.2				7.6	2.56	
Ekwunife et al.	2017	Nigeria	CRS	5010	108	2.7	2.77					
Okonkwo et al.	2011	Nigeria	CRS	1513	85	5.8	4.7	1.17				
Mashuda et al.	2014	Tanzania	CRS	445	131	6.1	2.3		3.81			
Muga et al.	2009	Kenya	Cohort	7355	207	7.2	2.9	1.5	0.4	1.45	0.96	
Shawky et al.	2011	Egypt	CRS	660,280	13543	24.5	18.6	0.31	0.26		1.50	1.05
Singh et al.	2015	Nigeria	CRS	10,163	72	1.38		1.38				
Waghathu et al.	2019	Kenya	CRS	315	61	1.63				1.63		
Nasio et al.	2020	Kenya	CRS	17,245	103	1.94	0.97					0.97
Abdou et al.	2019	Egypt	CCS	5710	5710	6.16						
ElAwady et al.	2019	Egypt	CRS	1000	74	10.8	9.7					1.35
AbouEl-Ella et al.	2018	Egypt	CRS	100	100	7						
Mohamed et al.	2011	Egypt	CRS	5000	103	27.1	17.4	1.9	0.97		4.85	1.94
El Koumi et al.	2013	Egypt	Cohort	2517	63	7.9	4.7	1.5			1.58	
Shalaby et al.	2020	Egypt	Cohort	346	173	1.15	1.15					
Afifi et al.	2011	Egypt	CRS	62,819	3417	9.3	7.3					
Forci et al.	2020	Morocco	CRS	43,923	245	16.3	14.2	0.4	0.81	0.81		
Abdu and Seyoum	2019	Ethiopia	CRS	22,624	310	2.58	2.5					
Ehianu et al.	2020	Nigeria	CRS	3171	203	3.4	3.4					
Ochoga et al.	2018	Nigeria	CRS	843	72	1.38						
Munkonge et al.	2007	Zambia	CRS	1,501,200	5478	0.98	0.45					0.34
Anyanwu et al.	2015	Nigeria	CRS	1456	41	4.87	2.4			2.4		
Kihshimba et al.	2015	Tanzania	CRS	28217	77	6.49	3.89	2.59				
Saib et al.	2021	S. Africa	CRS	7516	117	12.8	11.1	0.85	0.85			
Mkandawire et al.	2002	Malawi	CRS	9838	152	5.9	6					
Poaty et al.	2018	DRC	CRS	4785	430	19.3	19.3					
Christianson et al.	2002	S. Africa	CRS	6692	722	0.69	0.69					
Lebese et al.	2016	S. Africa	CRS	13252	13252	9.32	9.3					
Mombo	2017	Gabon	CRS	7712	32	3.1	3.12					
Kitova	2013	Tunisia	CRS	150	150	5.3	1.3		0.6	3.3		
Singh et al.	2000	Libya	CRS	16,186	151	23.1	21.8	1.32				
Farag et al.	2020	Libya	CRS	14,262	142	55.6	43.6	0.7				1.4
Ghareba et al.	2014	Libya	CRS	4850	73	8.2	8.2					
Haa EM et al.	2019	Libya	CRS	16765	98	41.8	32.7	4	5.1			1.02
Alkarshouf et al.	2019	Libya	CRS	81	81	48.1	48.1					
El-Sabbagh et al.	2009	Egypt	CRS	18,702	308	5.84	4.2					
Alburke et al.	2018	Libya	CRS	6239	73	5.47	1.3	4.1				
Abdellah et al.	2018	Sudan	CRS	2541	54	12.9	13					
Charlotte et al.	2015	Cameroon	CRS	6048	99	18.18	18.2					
Ajibola et al.	2017	Botswana	CRS	2944	33	3.0	3.03					
Zuechner et al.	2014	Tanzania	CRS	3982	1371	12.8	12.8					
Cavaliere et al.	2021	Mozambique	CRS	4767	143	11.18	7	0.7	3.5			
EL Madidi et al.	2021	Morocco	CCS	115	115	5.2						
Karim et al.	2019	Morocco	CRS	1010	81	7.4						
Elghanmi et al.	2020	Morocco	CRS	68704	706	12.3						
Abd Allah	2016	Sudan	CRS	2720	40	5	5					
Iroha et al.	2001	Nigeria	CRS	22288	353	2.83						
Mukhtar-Yola et al.	2005	Nigeria	CRS	182	75	5.3	2.6			2.6		

DRC-Democratic Republic of Congo, CRS-cross-sectional study, CCD-case control study, RCT-randomized control trial, CHR delet-chromosomal deletions, Turner-Turner syndrome, Un-class-unclassified chromosomal disorders, Chrom prop-proportion of chromosomal disorders, Down/T-21 = Down syndrome or Trisomy 21, Edwards'/T-18 = Edwards' syndrome or Trisomy-18, and Patau/T-13 = Patau syndrome or Trisomy 13.

**Table 2:** Subgroup analysis on the pooled proportion and patterns of chromosomal disorders among births with congenital anomalies: by country, region, and year of publication in Africa from January, 2000 to October, 2021.

Variables	Characteristics	Pooled prevalence	*I* ^2^ (*P* value)
By country	Libya	30.12 (13.17, 47.07)	96.9% (<0.001)
	DRC	19.3 (15.5, 23.03)	—
	Cameroon	18.18 (10.58, 23.7)	—
	Egypt	10.91 (4.04, 17.76)	99.5% (<0.001)
	Morocco	10.41 (5.8, 14.8)	80.7% (0.001)

By region	Central Africa	19.08% (95% CI: 15.74, 22.43)	—
	North Africa	15.06% (95% CI: 10.65, 19.47)	98.9 (<0.001)
	Southern Africa	5.4.0% (95% CI: 2.01, 8.79)	99% (<0.001)
	East Africa	4.880% (95% CI: 2.56, 7.20)	91.1% (<0.001)
	West Africa	4.03% (95% CI: 2.88, 5.18)	38.7% (=0.05)

By year of publication	2000–2014	8.32 (95% CI: 4.23, 12.15)	99.5% (*p* < 0.001)
	2015–2021	8.53% (95% CI: 6.95, 10.12)	93.8% (*p* < 0.001)

DRC-Democratic Republic of Congo.

**Table 3:** Pooled proportion and patterns of chromosomal disorders among births with congenital anomalies in Africa from January, 2000 to October, 2021.

Variables	Pooled proportion (95% CI)	*I* ^2^%	*P* value
Chromosomal disorders	8.94 (7.02, 10.86)	98.8	<0.001
Down syndrome	7.6 (5.70, 9.67)	98.9	<0.001
Edwards' syndrome	0.94 (0.49, 1.40)	31.2	0.11
Patau syndrome	0.92 (0.34, 1.51)	49.7	0.03
Turner syndrome	1.51 (1.3, 1.71)	—	0.56
Unclassified chromosomal disorders^*∗*^	1.78 (0.97, 2.60)	—	0.36
Chromosomal deletions	0.92 (0.37, 1.47)	82.3	<0.001

^*∗*^Unclassified chromosomal disorders—others and unclassified chromosomal anomalies.

## Data Availability

The data used to support the findings of this study are available and they can be accessed from the corresponding author when asked with a reasonable inquiry.

## Abbreviations

CAs: Congenital anomalies
T13: Trisomy 13
T18: Trisomy 18
T21: Trisomy 21.

## Supplementary Materials

Supplementary Materials Figure S1: Sensitivity analysis on the pooled proportion of chromosomal disorders among births with congenital anomalies in Africa from January, 2000 to October, 2021. Figure S2: Funnel plot on the pooled proportion of chromosomal disorders among births with congenital anomalies in Africa from January, 2000 to October, 2021. Figure S3. Forest plot on the pooled proportion of Down syndrome (Trisomy, 21) among births with congenital anomalies in Africa from January, 2000 to October, 2021. Figure S4: Forest plot on the pooled proportion of Edwards' syndrome (Trisomy, 18) among births with congenital anomalies in Africa from January, 2000 to October, 2021. Figure S5: Forest plot on the pooled proportion of Patau Syndrome (Trisomy, 13) among births with congenital anomalies in Africa from January, 2000 to October, 2021. Figure S6: Forest plot on the pooled proportion of Turner syndrome among births with congenital anomalies in Africa from January, 2000 to October, 2021. Figure S7: Forest plot on the pooled proportion of chromosomal deletions among births with congenital anomalies in Africa from January, 2000 to October, 2021. Figure S8: Forest plot on the pooled proportion of unclassified chromosomal disorders among births with congenital anomalies in Africa from January, 2000 to October, 2021.
