# Supplementary material for: Proportion of Chromosomal Disorders and Their Patterns among Births with Congenital Anomalies in Africa: A Systematic Review and Meta-Analyses
Source: ScientificWorldJournal. 2022 Dec 13;2022:6477596. doi: 10.1155/2022/6477596 (PMC9767725; doi:10.1155/2022/6477596)
Supplement: Supplementary Materials — Figure S1: Sensitivity analysis on the pooled proportion of chromosomal disorders among births with congenital anomalies in Africa from January, 2000 to October, 2021. Figure S2: Funnel plot on the pooled proportion of chromosomal disorders among births with congenital anomalies in Africa from January, 2000 to October, 2021. Figure S3. Forest plot on the pooled proportion of Down syndrome (Trisomy, 21) among births with congenital anomalies in Africa from January, 2000 to October, 2021. Figure S4: Forest plot on the pooled proportion of Edwards' syndrome (Trisomy, 18) among births with congenital anomalies in Africa from January, 2000 to October, 2021. Figure S5: Forest plot on the pooled proportion of Patau Syndrome (Trisomy, 13) among births with congenital anomalies in Africa from January, 2000 to October, 2021. Figure S6: Forest plot on the pooled proportion of Turner syndrome among births with congenital anomalies in Africa from January, 2000 to October, 2021. Figure S7: Forest plot on the pooled proportion of chromosomal deletions among births with congenital anomalies in Africa from January, 2000 to October, 2021. Figure S8: Forest plot on the pooled proportion of unclassified chromosomal disorders among births with congenital anomalies in Africa from January, 2000 to October, 2021. [file 6477596.f1.zip › supplementary figures (1).docx]

Figure S1: Sensitivity analysis on the pooled proportion of chromosomal disorders among births with congenital anomalies in Africa from January, 2000-October, 2021.


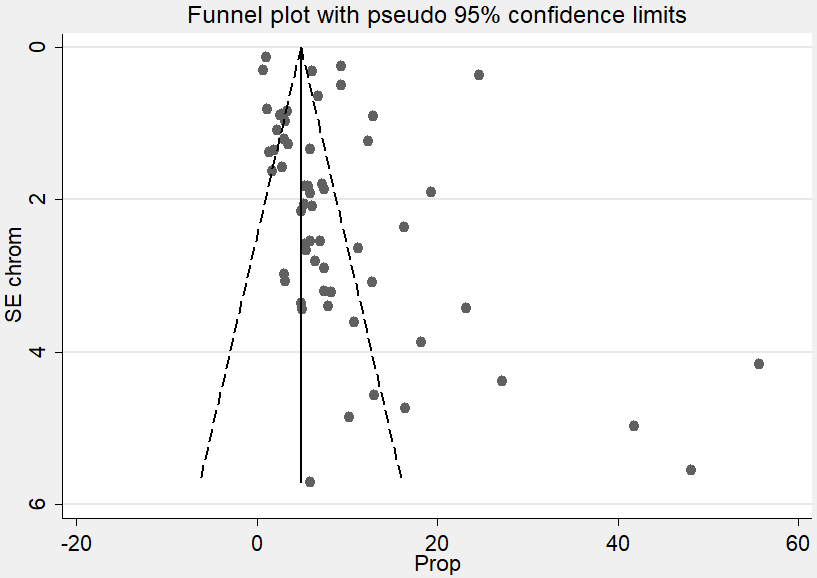


Figure S2: Funnel plot on the pooled proportion of chromosomal disorders among births with congenital anomalies in Africa from January 2000-October, 2021.

Figure S3. Forest plot on the pooled proportion of Down syndrome (Trisomy, 21) among births with congenital anomalies in Africa from January 2000-October, 2021


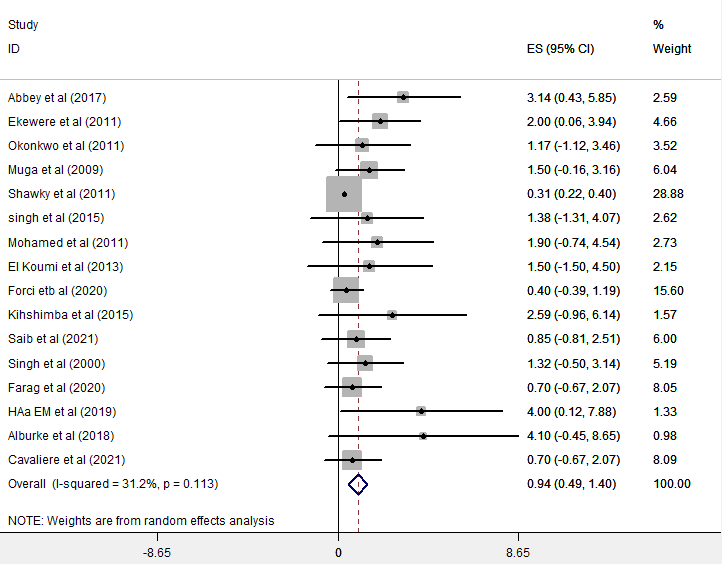


Figure S4: Forest plot on the pooled proportion of Edwards Syndrome (Trisomy, 18) among births with congenital anomalies in Africa from January 2000-October, 2021


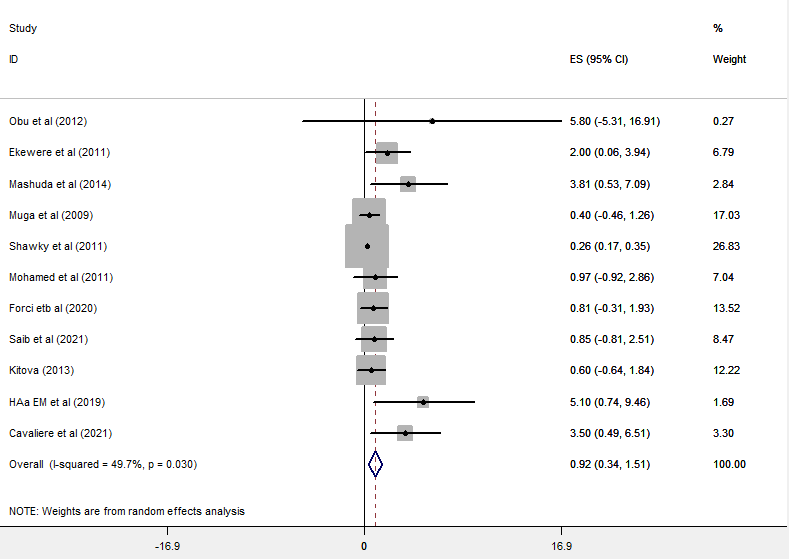


Figure S5: Forest plot on the pooled proportion of Patau Syndrome (Trisomy, 13) among births with congenital anomalies in Africa from January 2000-October, 2021


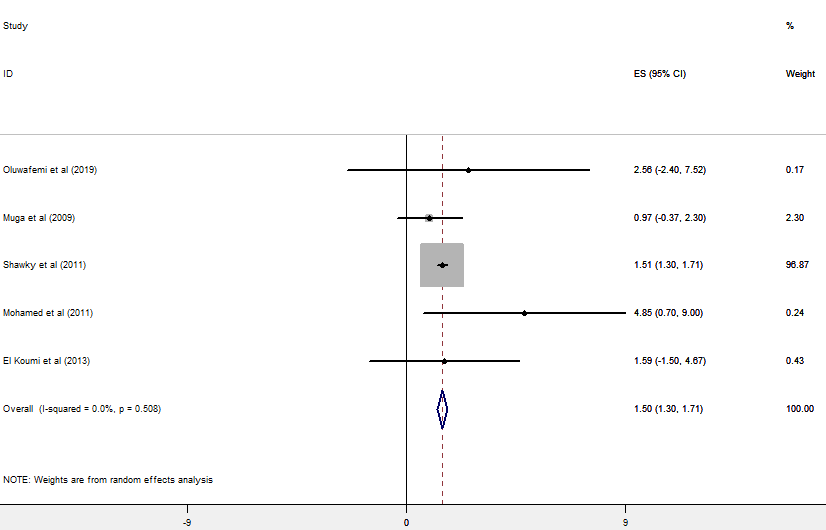


Figure S6: Forest plot on the pooled proportion of Turners syndrome among births with congenital anomalies in Africa from January 2000-October, 2021


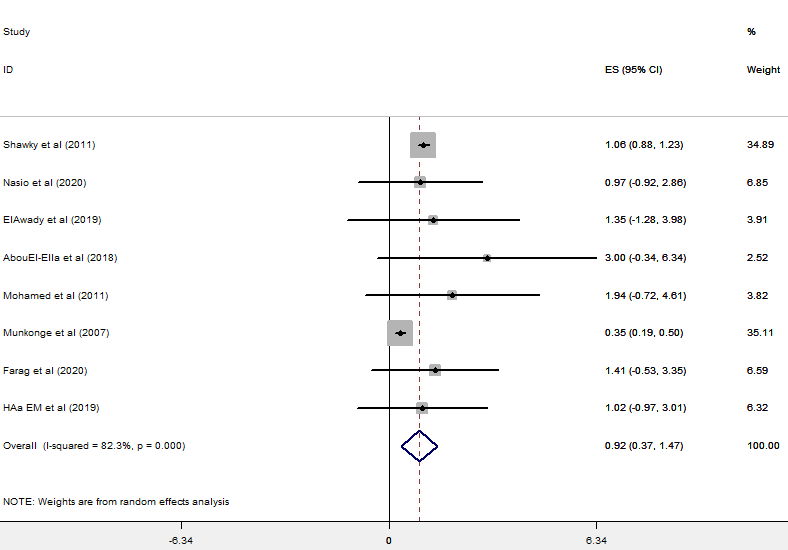


Figure S7: Forest plot on the pooled proportion of chromosomal deletions among births with congenital anomalies in Africa from January 2000-October, 2021


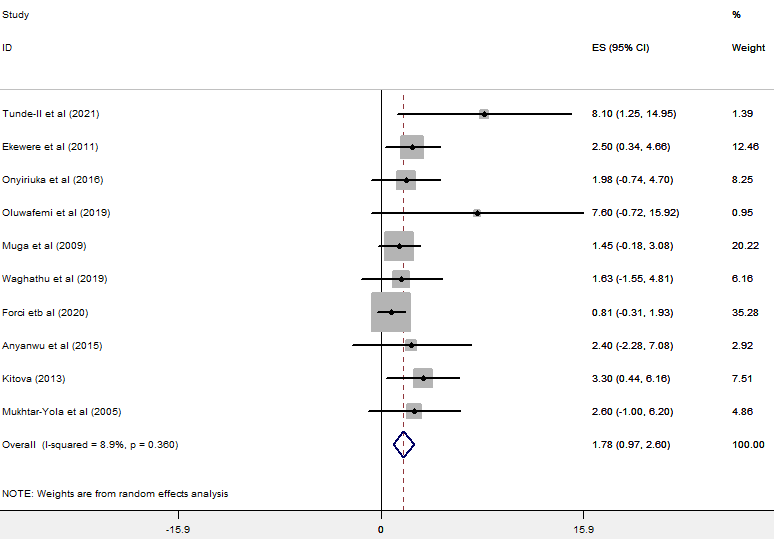


Figure S8: Forest plot on the pooled proportion of unclassified chromosomal disorders among births with congenital anomalies in Africa from January 2000-October, 2021
