# Supplementary material for: Proportion of Chromosomal Disorders and Their Patterns among Births with Congenital Anomalies in Africa: A Systematic Review and Meta-Analyses
Source: ScientificWorldJournal. 2022 Dec 13;2022:6477596. doi: 10.1155/2022/6477596 (PMC9767725; doi:10.1155/2022/6477596)
Supplement: Supplementary Materials — Figure S1: Sensitivity analysis on the pooled proportion of chromosomal disorders among births with congenital anomalies in Africa from January, 2000 to October, 2021. Figure S2: Funnel plot on the pooled proportion of chromosomal disorders among births with congenital anomalies in Africa from January, 2000 to October, 2021. Figure S3. Forest plot on the pooled proportion of Down syndrome (Trisomy, 21) among births with congenital anomalies in Africa from January, 2000 to October, 2021. Figure S4: Forest plot on the pooled proportion of Edwards' syndrome (Trisomy, 18) among births with congenital anomalies in Africa from January, 2000 to October, 2021. Figure S5: Forest plot on the pooled proportion of Patau Syndrome (Trisomy, 13) among births with congenital anomalies in Africa from January, 2000 to October, 2021. Figure S6: Forest plot on the pooled proportion of Turner syndrome among births with congenital anomalies in Africa from January, 2000 to October, 2021. Figure S7: Forest plot on the pooled proportion of chromosomal deletions among births with congenital anomalies in Africa from January, 2000 to October, 2021. Figure S8: Forest plot on the pooled proportion of unclassified chromosomal disorders among births with congenital anomalies in Africa from January, 2000 to October, 2021. [file 6477596.f1.zip › supplementary figures-.pdf]

Study omitted	Estimate	[95% Conf.	Interval]
1	9.0552406	7.1033344	11.007148
2	8.9962721	7.0294671	10.963077
3	9.0544472	7.1066952	11.002199
4	9.0343199	7.0951576	10.973483
5	9.0551481	7.0989285	11.011368
6	8.9641027	7.0253143	10.90289
7	9.0704184	7.1215038	11.019333
8	9.000844	7.0577192	10.943969
9	8.84126	6.906549	10.775971
10	8.9757643	7.041688	10.909841
11	8.967577	7.0249238	10.91023
12	9.0113268	7.0695229	10.953132
13	8.9230976	6.9877601	10.858436
14	9.0547943	7.1105013	10.999088
15	8.9928493	7.0523167	10.933383
16	8.9914112	7.049427	10.933396
17	8.9723978	7.0294032	10.915393
18	8.0547733	6.7230206	9.3865252
19	9.0820007	7.1368995	11.027102
20	9.0747786	7.1311755	11.018382
21	9.0722427	7.1266356	11.017849
22	9.0568504	7.0016909	11.112009
23	8.9118996	6.9743643	10.849435
24	8.9738808	7.0334435	10.914317
25	8.6854811	6.7537031	10.617258
26	8.9561625	7.0178413	10.894484
27	9.0948296	7.1405787	11.049082
28	8.9512053	6.9789171	10.923493
29	8.811655	6.874783	10.748528
30	9.067296	7.1137009	11.02089
31	9.0457144	7.0986338	10.992794
32	9.0820007	7.1368995	11.027102
33	9.1280079	7.1233306	11.132686
34	9.0040636	7.0655036	10.942623
35	8.9813299	7.0415149	10.921145
36	8.877739	6.9397497	10.815728
37	8.9956856	7.0529637	10.938408
38	8.7508888	6.818481	10.683296
39	9.1456604	7.1180243	11.173296
40	8.9990444	6.9404798	11.057609
41	9.0343199	7.0951576	10.973483
42	9.0067024	7.0635648	10.94984
43	8.7171831	6.7839108	10.650456
44	8.2639122	6.35394	10.173884
45	8.9520473	7.0133467	10.890747
46	8.5143785	6.5889864	10.439771
47	8.4704933	6.5471292	10.393857
48	9.000967	7.0543885	10.947545
49	8.9990005	7.0587559	10.939245
50	8.885725	6.9501958	10.821254
51	8.8032541	6.8679171	10.738592
52	9.0367746	7.0974026	10.976147
53	8.8666258	6.9262738	10.806978
54	8.9026699	6.9633799	10.84196
55	9.0075321	7.0654058	10.949659
56	8.9659977	7.0265117	10.905484
57	8.8783045	6.9370365	10.819571
58	9.0015812	7.0632024	10.93996
59	9.0631418	7.108747	11.017535
60	9.0024977	7.0620384	10.942957
61	8.9399633	7.0175343	10.862392
62	8.9399633	7.0175343	10.862392
63	8.9399633	7.0175343	10.862392
64	8.9399633	7.0175343	10.862392
65	8.9399633	7.0175343	10.862392
66	8.9399633	7.0175343	10.862392
67	8.9399633	7.0175343	10.862392
Combined	8.9399635	7.0175343	10.862393

Figure S1: Sensitivity analysis on the pooled proportion of chromosomal disorders among births with congenital anomalies in Africa from January, 2000-October, 2021.

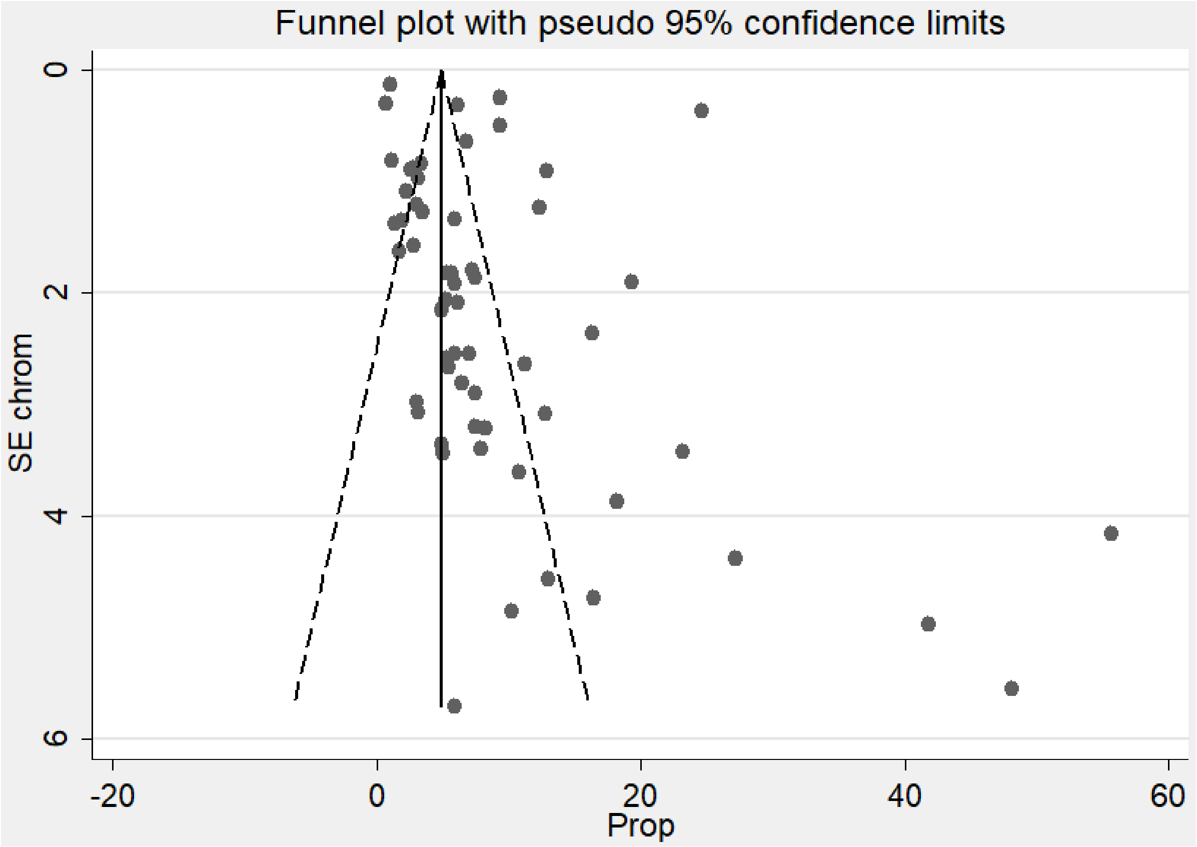

Figure S2: Funnel plot on the pooled proportion of chromosomal disorders among births with congenital anomalies in Africa from January 2000-October, 2021.

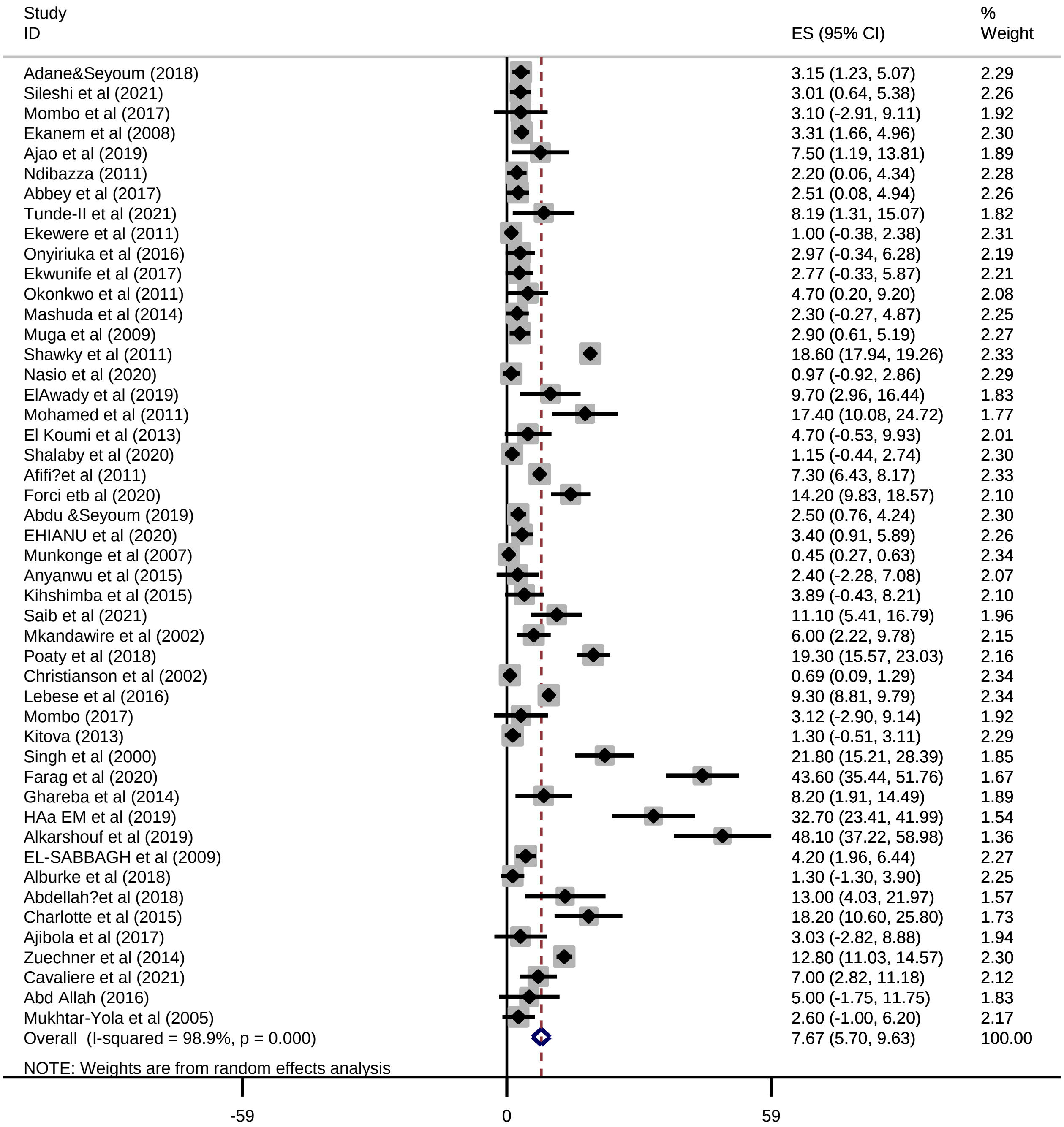

Figure S3. Forest plot on the pooled proportion of Down syndrome (Trisomy, 21) among births with congenital anomalies in Africa from January 2000-October, 2021

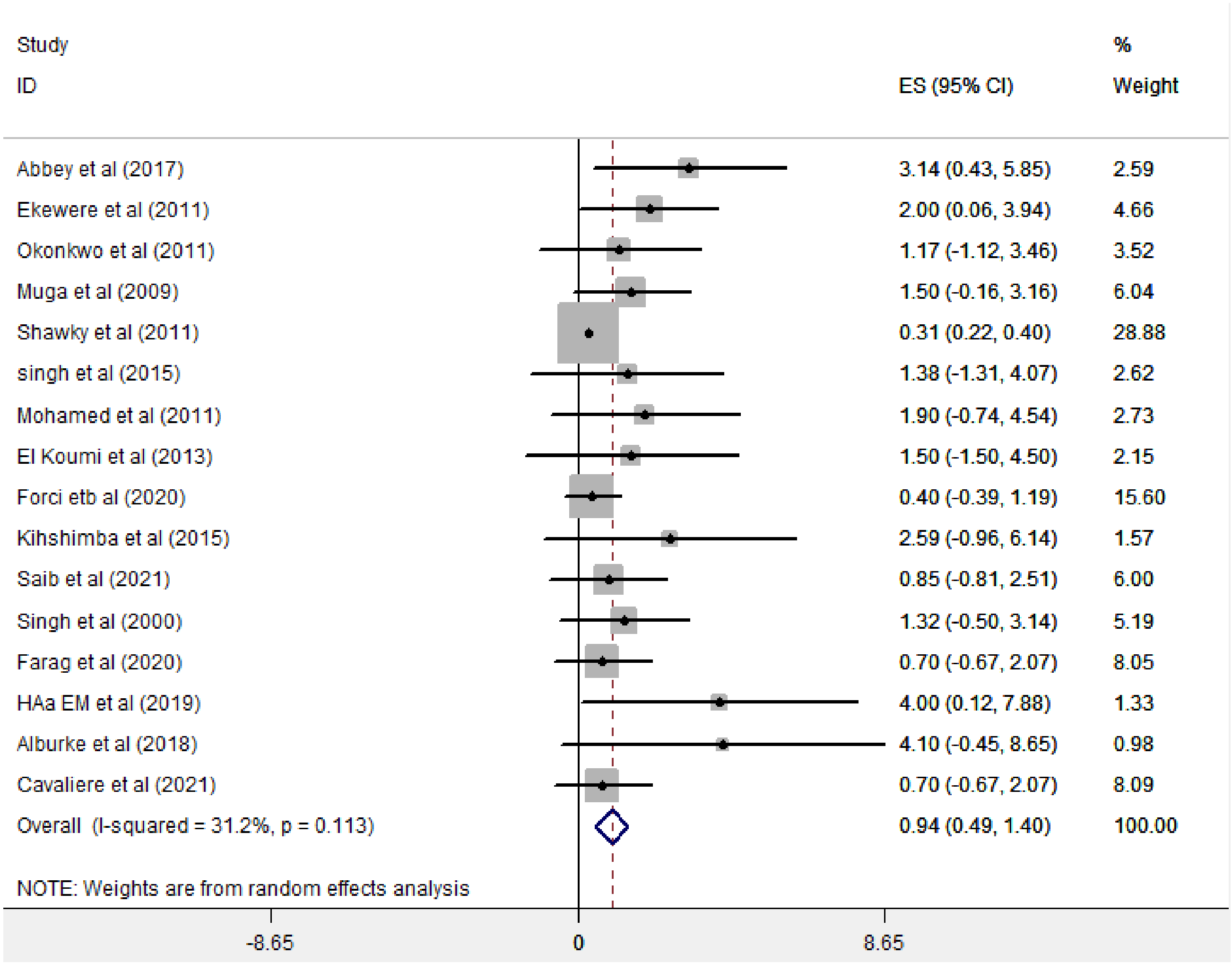

Figure S4: Forest plot on the pooled proportion of Edwards Syndrome (Trisomy, 18) among births with congenital anomalies in Africa from January 2000-October, 2021

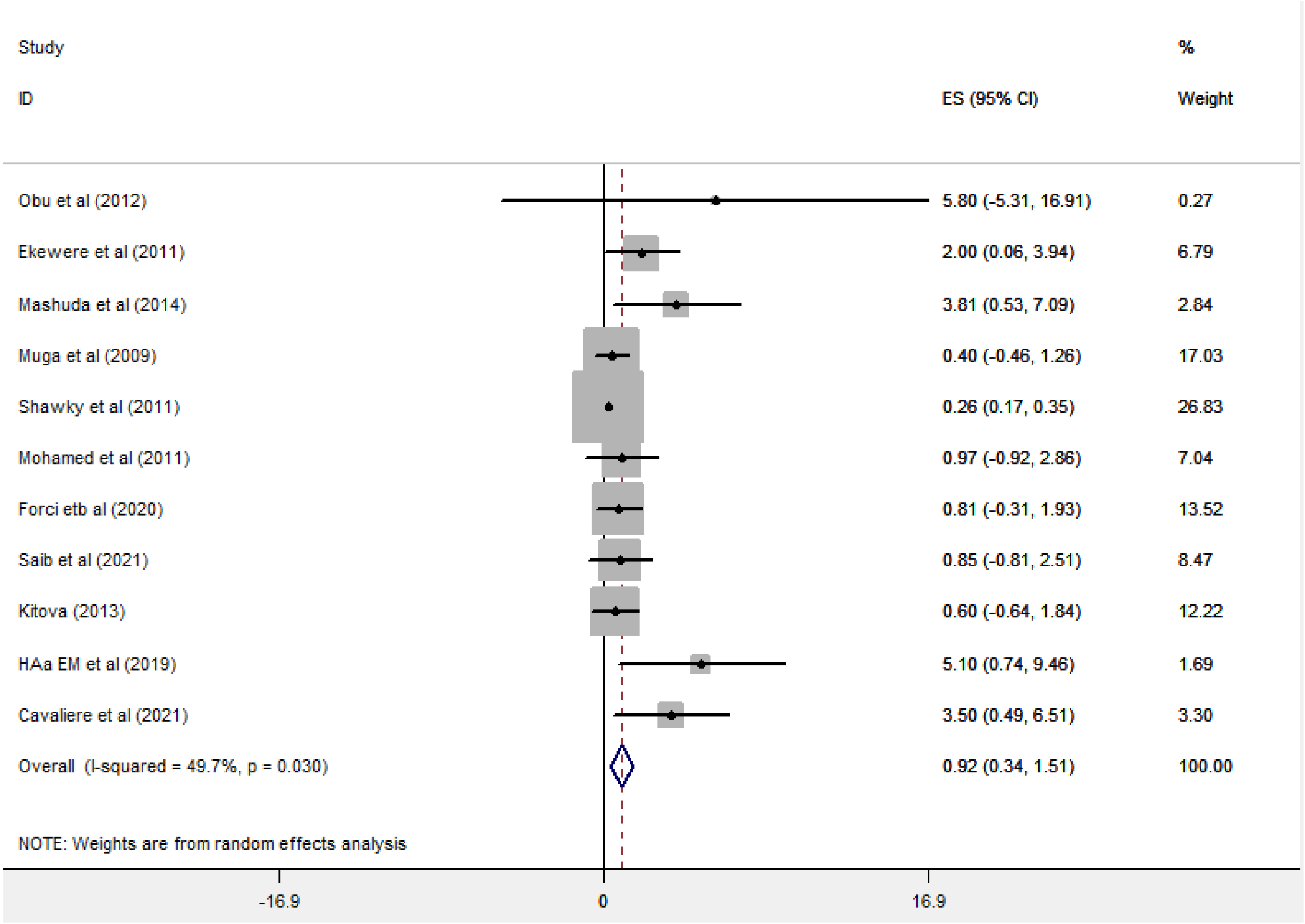

Figure S5: Forest plot on the pooled proportion of Patau Syndrome (Trisomy, 13) among births with congenital anomalies in Africa from January 2000-October, 2021

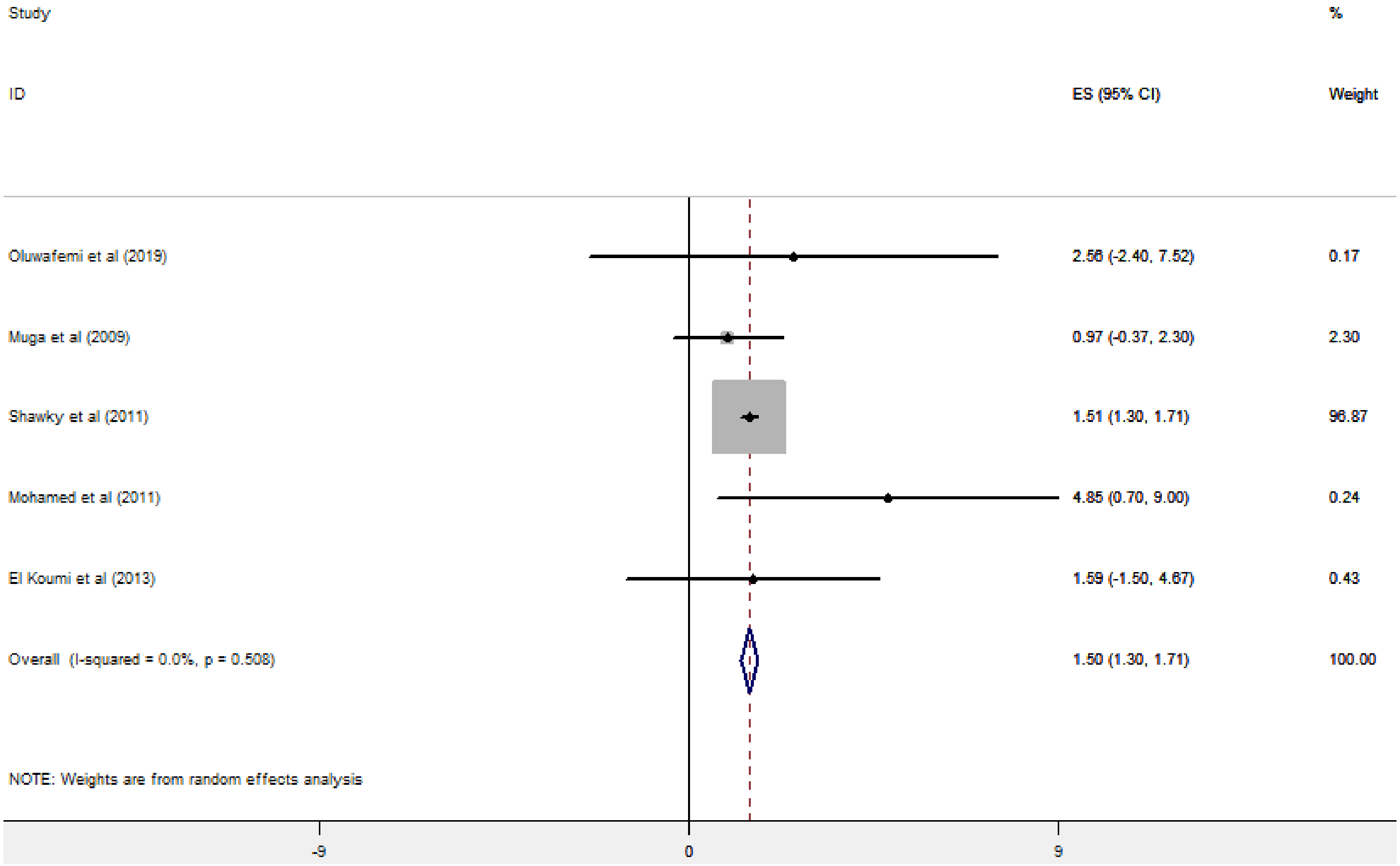

Figure S6: Forest plot on the pooled proportion of Turners syndrome among births with congenital anomalies in Africa from January 2000-October, 2021

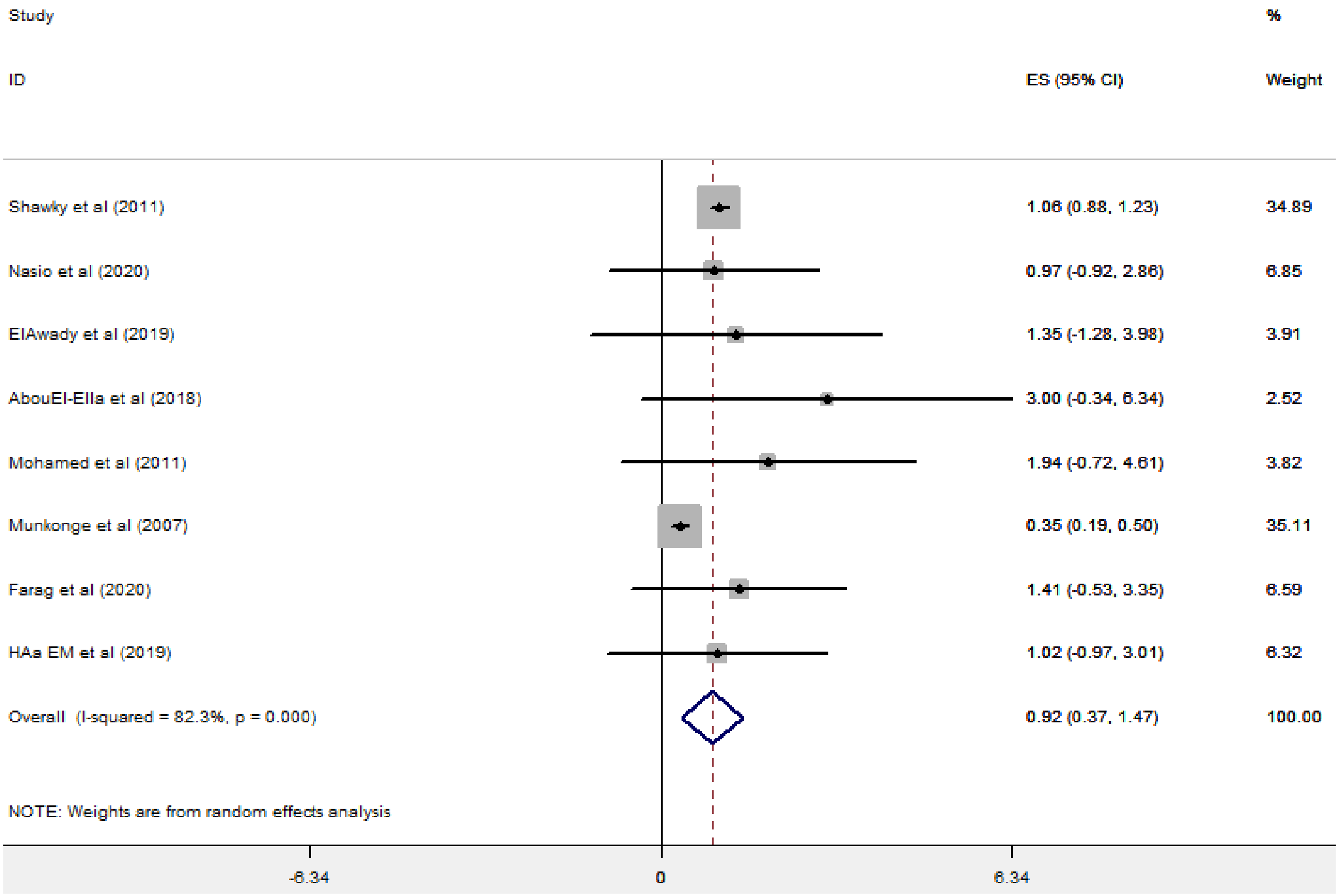

Figure S7: Forest plot on the pooled proportion of chromosomal deletions among births with congenital anomalies in Africa from January 2000-October, 2021

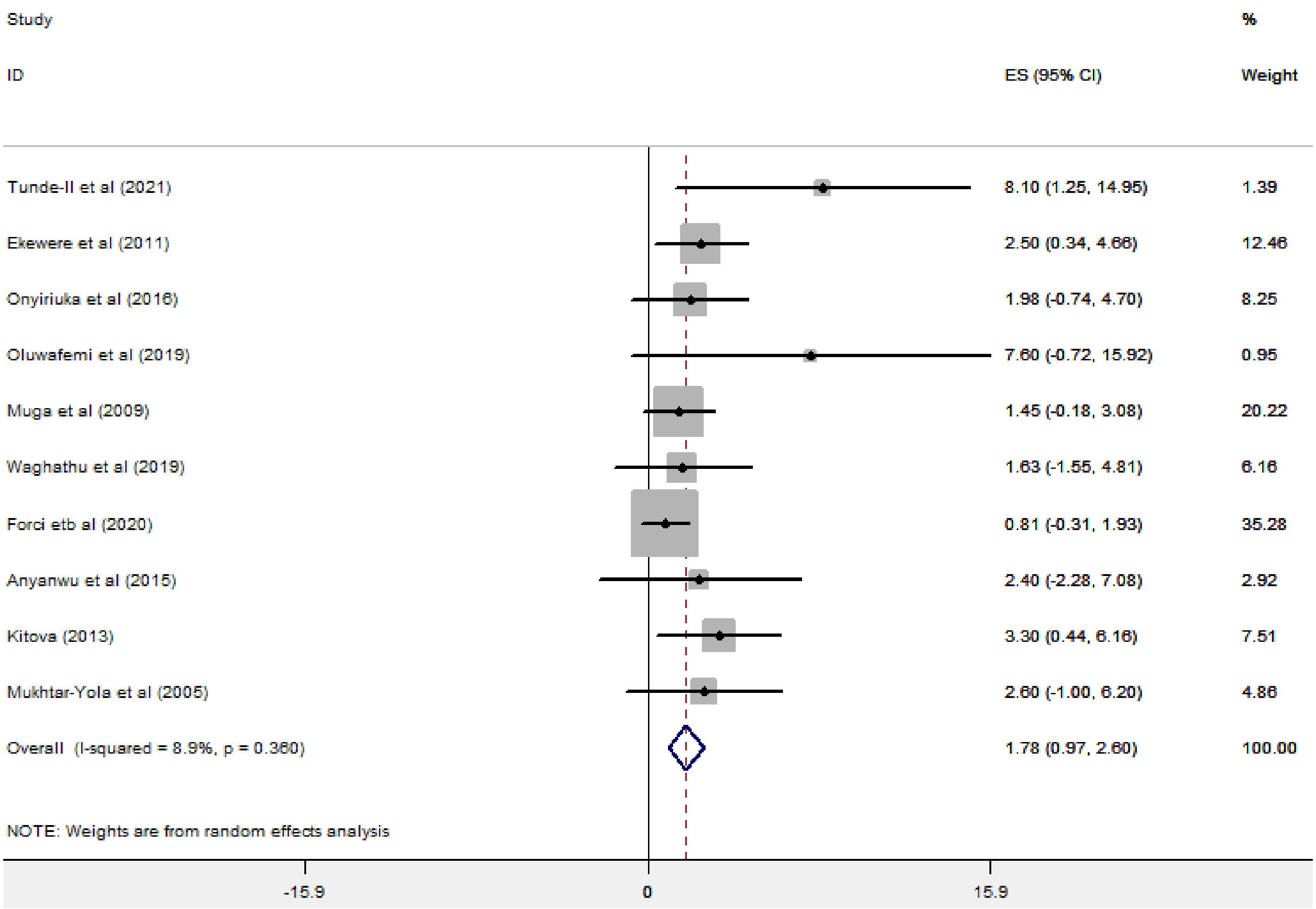

Figure S8: Forest plot on the pooled proportion of unclassified chromosomal disorders among births with congenital anomalies in Africa from January 2000-October, 2021
